# The Effect of Boron (B) and Copper (Cu) on the Microstructure and Graphite Morphology of Spheroidal Graphite Cast Iron

**DOI:** 10.3390/ma16124225

**Published:** 2023-06-07

**Authors:** Jin-Su Ha, Ji-Woo Hong, Ji-Wook Kim, Soo-Bin Han, Chang-Young Choi, Hye-Jin Song, Jin-Seok Jang, Dong-Yul Kim, Dae-Cheol Ko, Seong-Hoon Yi, Yong-Jae Cho

**Affiliations:** 1 Smart Manufacturing Technology R&D Group, Korea Institute of Industrial Technology, Daegu 42994, Republic of Korea; jinsu302@kitech.re.kr (J.-S.H.); wldn4116@kitech.re.kr (J.-W.H.); jwkim0@kitech.re.kr (J.-W.K.); soobin35@kitech.re.kr (S.-B.H.); ccy8584@kitech.re.kr (C.-Y.C.); shyejin@kitech.re.kr (H.-J.S.); jsjang@kitech.re.kr (J.-S.J.); 2 Gyeongbuk Research Institute of Vehicle Embedded Technology, Yeongcheon-si 38822, Republic of Korea; dykim@givet.re.kr; 3 Department of Nanomechatronics Engineering, Pusan National University, Pusan 46241, Republic of Korea; dcko@pusan.ac.kr; 4 Department of Materials Science and Metallurgical Engineering, Kyungpook National University, Daegu 41566, Republic of Korea

**Keywords:** spheroidal graphite cast iron, boron, pearlite, mechanical properties, microstructure

## Abstract

This study examines the impacts of copper and boron in parts per million (ppm) on the microstructure and mechanical properties of spheroidal graphite cast iron (SCI). Boron’s inclusion increases the ferrite content whereas copper augments the stability of pearlite. The interaction between the two significantly influences the ferrite content. Differential scanning calorimetry (DSC) analysis indicates that boron alters the enthalpy change of the α + Fe3C → γ conversion and the α → γ conversion. Scanning electron microscope (SEM) analysis confirms the locations of copper and boron. Mechanical property assessments using a universal testing machine show that the inclusion of boron and copper decreases the tensile strength and yield strength of SCI, but simultaneously enhances elongation. Additionally, in SCI production, the utilization of copper-bearing scrap and trace amounts of boron-containing scrap metal, especially in the casting of ferritic nodular cast iron, offers potential for resource recycling. This highlights the importance of resource conservation and recycling in advancing sustainable manufacturing practices. These findings provide critical insights into the effects of boron and copper on SCI’s behavior, contributing to the design and development of high-performance SCI materials.

## 1. Introduction

In recent years, the requirements for automobile parts include high-strength and high-elongation characteristics that can compete with forged steel or austempered ductile iron (ADI) materials, all while maintaining cost competitiveness. Therefore, industrially, the production and practical application research of spheroidal graphite cast iron, which requires high strength and high elongation, is in a very active state. Particularly, research on optimal mechanical characteristics implementation due to the addition of alloy elements is considered highly important [1,2,3]. 

As such, casting is still widely used in various fields today. Metal has high deformation resistance, so it is difficult to mold it into a desired shape. By using casting, metals can be melted into a low-deformation liquid state and solidified into the desired shape, making it possible to create complex shapes all at once. In addition, compared to other manufacturing techniques, casting has simpler processes and requires less post-treatment, making it a viable option in the recent electric car market. Metals suitable for casting possess certain characteristics. First, they have a low melting point, which makes them easier to work with as they require less energy to transition from a solid to a liquid state. Second, when melted, these metals exhibit good fluidity. This is crucial because it allows the molten metal to flow and fill the mold accurately, capturing all the necessary details. Lastly, these metals exhibit minimal shrinkage during the process of solidification. Low shrinkage is important to maintain the desired shape and dimensions of the cast object, as high shrinkage can lead to deformations and discrepancies in the final product [4,5]. 

Cast iron satisfies the above conditions and allows for the relatively easy production of complex shapes. Compared to other metals, cast iron is relatively inexpensive and offers various mechanical properties. Cast iron typically exhibits a flake graphite morphology, where the graphite forms thin, flat flakes within the matrix. This flake-like structure can introduce areas of stress concentration, reducing the overall strength and ductility of the material. On the other hand, spheroidal graphite cast iron, as the name suggests, features graphite nodules that have a spherical or nodular shape. This unique morphology effectively distributes stress throughout the material, enhancing its mechanical properties such as strength and ductility. The distinct morphological differences between these two types of iron alloys contribute to their contrasting performance characteristics and suitability for various applications. Spheroidal graphite cast iron contains a large amount of added alloy elements, has a low melting point and excellent castability, and the mechanical properties can be controlled by adjusting the ratio of alloy elements. It is widely used in various fields, such as automobile parts, machine parts, and materials for rolling mills, as well as for parts requiring heat resistance [4,5].

Following the revival of interest in the mechanism of SG formation due to the advancements in equipment and electron microscopy techniques, it is important to note the significance of cast iron [6].

The mechanical properties of spheroidal graphite cast iron are determined by the shape of graphite, the number of graphite nodules, and microstructure. In particular, the mechanical properties of spheroidal graphite cast iron can change significantly depending on the ratio of ferrite to pearlite, and to control these microstructural properties, it is necessary to control the cooling rate, the type and amount of alloy elements, and other process variables [7,8,9]. 

In the solidification and heating processes of cast iron, carbon diffusion plays a crucial role in determining the microstructural evolution and mechanical properties of the material. During solidification, as the molten iron cools and transforms into a solid, carbon atoms migrate and diffuse within the austenite matrix. This carbon diffusion process is responsible for the formation of various microstructural constituents, such as pearlite and ferrite, which significantly influence the material’s mechanical behavior. Furthermore, in the heating process, as cast iron is subjected to elevated temperatures, carbon diffusion continues to occur. This time, carbon atoms diffuse out of the graphite phase and into the austenite matrix, resulting in phase transformations and changes in the material’s microstructure. Understanding the mechanisms and kinetics of carbon diffusion during the solidification and heating processes is essential for controlling the microstructural properties and tailoring the mechanical performance of cast iron. It enables us to optimize casting conditions, alloy compositions, and heat treatment procedures to achieve desired material characteristics [10,11]. 

This study investigated the effect of B, an alloy element commonly added during the casting of iron products, on the microstructure of cast iron. When casting, pig iron and steel scrap are mixed together, and various alloy elements are added to the steel scrap to improve the quality of iron products. Steel scrap from automobile steel plates is commonly used in casting. High-strength steel plates used for automobile panels contain B as an alloy element, which improves the hardenability of steel. By adding B, expensive alloy elements can be replaced, and high-strength steel can be produced at a lower cost. When B is added to cast iron, it affects the microstructure. Recent studies have reported that adding small amounts of B at the ppm level stabilizes ferrite in cast iron [12,13,14]. 

In addition, there is research that shows the ferrite ratio increases by analyzing the microstructure characteristics for a small amount of boron. When this phenomenon occurs in pearlite cast iron, it is reported that the microstructure of the casting changes and the quality of the product deteriorates [15]. 

Therefore, in this study, we chose pearlitic spheroidal graphite cast iron, which is extensively used in diverse domains such as automobiles and ships. We adjusted the ratios of Mn and Cu to control the pearlite ratio in the specimens. Diverging from previous research, we broadened our investigation to include not just microstructure analysis but also mechanical property tests and heat analysis on the fabricated specimens. The influence of small amounts of B on spheroidal graphite cast iron was analyzed based on these comprehensive results. The outcomes of this study will be utilized for the manufacture of ferritic nodular cast iron, leveraging pearlite reinforcing elements and boron.

## 2. Materials and Methods

### 2.1. Melting and Casting

The melting of cast iron was achieved using high-frequency induction melting (350PT10, HanGuk Induction) to melt the alloying elements, and the composition of the target chemical components is shown in Table 1. The nodularization of graphite was achieved by adding Mg-Re(Si 45.11%, Mg 5.62%, Ca 2.16%, Al 1.94%, Re 1.94%) nodularizing agent using the sandwich method and adding inoculant when ladling. The amount of B was controlled by using Fe-B(C 0.31%, Si 0.5%, P 0.042%, S 0.004%, Al 0.122%, B 15.24%). The ladle tapping temperature from the furnace was 1450 °C, and the melt pouring temperature of the Y-block from the ladle was 1300 °C. After adding B to the molten iron, the molten iron was maintained for 6 min in the ladle before being tapped. Type 2 Y-block was used as specified in DIN EN 1563.

### 2.2. Mechanical Property Evaluation

Y-blocks were cast and used to produce tension and impact test specimens. The tension test specimen was made in the shape of a rod, which is one of the test specimens used in KS B 0801 metallic material tensile tests. The tensile strength, yield strength, and elongation were measured using a universal testing machine (Instron 5988, Instron, Norwood, MA, USA) and an extensometer (AUTOX 750, Instron, Norwood, MA, USA). The test was conducted at room temperature with a crosshead speed of 10 mm/min. The gauge length was fixed at 50 mm. The impact test was performed using a Charpy impact tester (Instron MPX-700, Instron, Norwood, MA, USA) with V-notch impact specimens produced according to KS B 0809.

### 2.3. Chemical Composition Analysis

A small amount of molten metal was collected before melting and pouring into the mold for chemical composition analysis to make a cast specimen. The analysis surface of the solidified cast specimen was ground with a grinder, and the chemical composition was analyzed using a Spark Optical Emission Spectrometer (OBLF GmbH QSN750-2, OBLF GMBH, Witten, Germany).

### 2.4. Microstructure and Graphite Analysis

During the production of the tension test specimens, cylindrical specimens with a diameter of approximately 22 mm were also made to observe the microstructure. The specimens were mechanically polished using an automatic polishing machine (Tegramin-25, Struers, Ballerup, Denmark), followed by polishing with 1μm diamond suspension and ethanol on a buffer. The specimens were then etched in a 3% nital solution for about 15 s, and the microstructure was observed using an optical microscope (Axio Observer. A1m, Carl Zeiss Microscopy GmbH, Jena, Germany) and measured for the percentage of graphite, graphite size, and nodularity using image analysis software (IMT iSolution DT ver.20.1). Additionally, the distribution of elements and the surface morphology of graphite were analyzed using a field-emission scanning electron microscope (FE-SEM, JSM-7900F AZtec Live and AZtec HKL, JEOL Ltd., Tokyo, Japan), depending on the presence or absence of B.

### 2.5. Thermal Analysis

The state change temperature and enthalpy of each specimen were measured to analyze the amount of reaction using differential thermal analysis (DTA). The heat flow difference between the reference pan and the specimen-containing pan was analyzed using a differential scanning calorimeter (TA Instruments SDT600, New Castle, DE, USA) with an alumina 90-uL crucible. The analysis was performed at a heating rate of 10 °C/min from 50 °C to 1300 °C.

## 3. Results and Discussion

### 3.1. Microstructure Analysis

**Figure 1:**
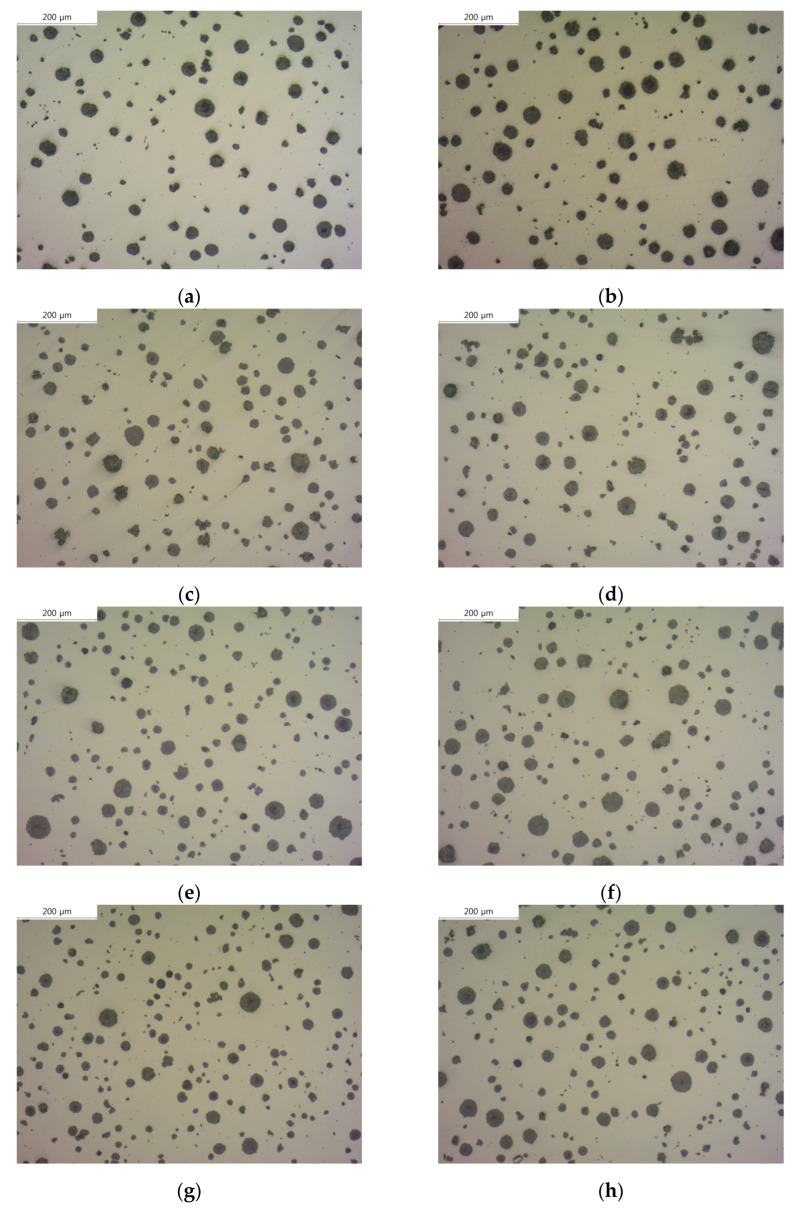
Microstructures of as-polished spheroidal graphite cast iron observed by an optical microscope: (**a**) SCI–Cu, (**b**) SCI–CuB, (**c**) SCI–Mn, (**d**) SCI–MnB, (**e**) SCI–Mn_0.5_Cu_0.5_, (**f**) SCI–Mn_0.5_Cu_0.5_B, (**g**) SCI–Mn_0.5_Cu_1_, and (**h**) SCI–Mn_0.5_Cu_1_B.

Figure 1 shows the microstructure images of each specimen before etching with and without the addition of B. Using these images, we measured the graphite size, graphite nodule count, and graphite area using an image analysis program (IMT iSolution DT) and listed the results in Table 2. The graphite size of specimens with B added tended to increase, up to a maximum of 17%. In contrast, the graphite nodule count decreased up to a maximum of 22%, which is contrary to the trend of the graphite size. Despite the decrease in the graphite nodule count and increase in the graphite size, the total graphite area increased, suggesting that carbon diffusion from austenite to graphite occurred.

**Figure 2:**
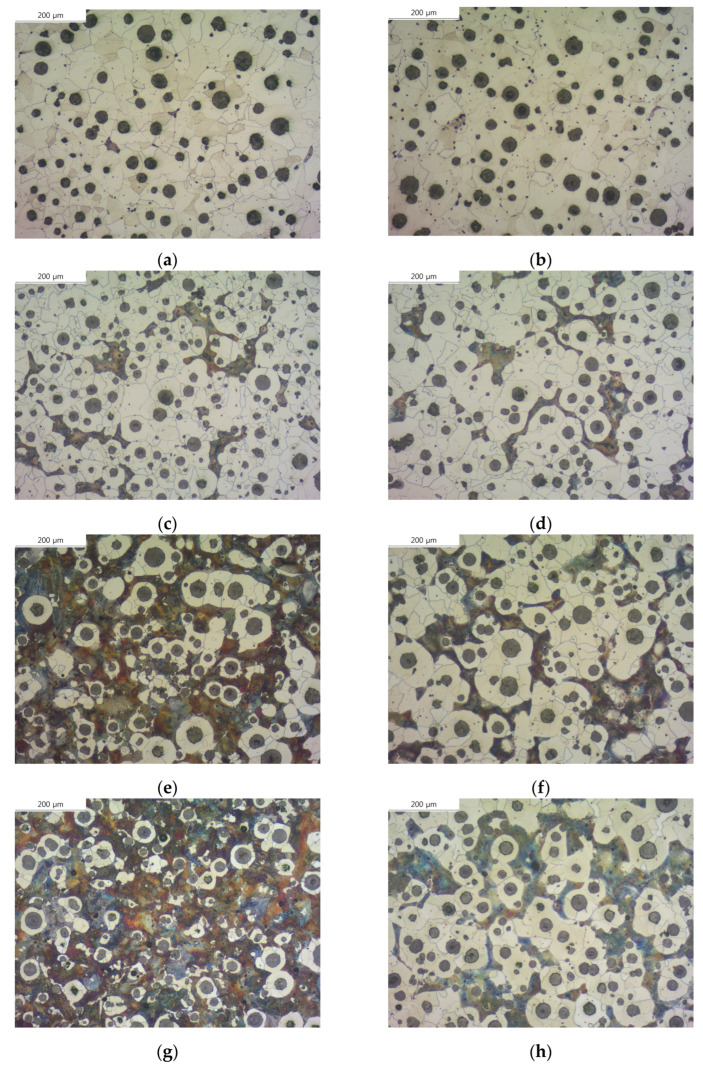
Microstructures of etched spheroidal graphite cast iron observed by an optical microscope: (**a**) SCI–Cu, (**b**) SCI–CuB, (**c**) SCI–Mn, (**d**) SCI–MnB, (**e**) SCI–Mn_0.5_Cu_0.5_, (**f**) SCI–Mn_0.5_Cu_0.5_B, (**g**) SCI–Mn_0.5_Cu_1_, and (**h**) SCI–Mn_0.5_Cu_1_B.

Figure 2 shows the images of each specimen after etching, and we measured the ferrite area fraction of each specimen. Previous studies have shown that a small amount of Cu neutralizes the effect of other ferritic alloying elements, and a certain amount of Cu is necessary to act as a strong ferrite-forming element [13,16,17,18]. In our experiment, specimens with both Cu and Mn added formed more ferrite than specimens with only Cu or Mn added, and the ferrite area of the SCI–Cu specimen was almost negligible (6.5%). On the other hand, the ferrite area of the SCI–Mn_0.5_Cu_1_ specimen increased about three times compared to that of specimens with only Cu or Mn added. From Figure 2 and Table 2, we can observe that the ferrite area fraction of specimens with B added increased.

Through optical microscopy, we confirmed that the addition of B affects graphite and ferrite. By comparing the images of corroded specimens in Figure 2 and the data in Table 2, we concluded that B has a greater influence on the ferrite transformation in pearlitic cast iron.

### 3.2. Comparison of Graphite Shape

**Figure 3:**
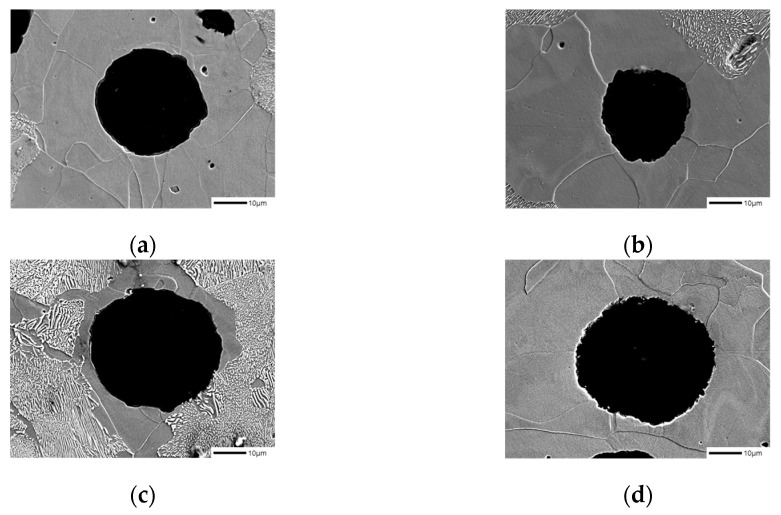
Microstructures of samples etched with 3% nital observed by SEM: (**a**) SCI–Mn_0.5_Cu_0.5_, (**b**) SCI–Mn_0.5_Cu_0.5_B, (**c**) SCI–Mn_0.5_Cu_1_, and (**d**) SCI–Mn_0.5_Cu_1_B.

When comparing the microstructure of Mn_0.5_Cu_0.5_ and Mn_0.5_Cu_1_ specimens with a significant change in the ferrite fraction after being corroded in 3% nital for 10 min, we found that there is a difference in the surface shape of graphite before and after the addition of B, as shown in Figure 3a–d [15].

**Figure 4:**
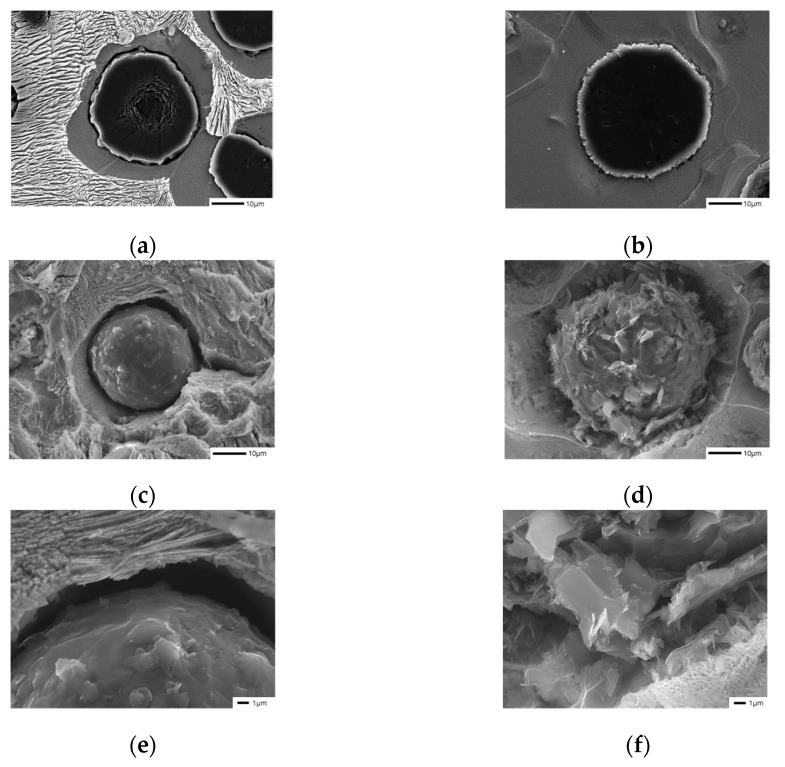
Graphite shapes of samples deep-etched with 3% nital observed by SEM: (**a**,**c**,**e**) SCI–Mn_0.5_Cu_1_; (**b**,**d**,**f**) SCI–Mn_0.5_Cu_1_B.

We observed the surface of graphite, which was not very visible in the cross-section, by preparing a polished surface specimen in the same way as the microstructure specimen (Figure 4c–d). The surface area of graphite increased due to the addition of B, and this is expected to reduce the effect of the Cu film generated by the graphite precipitation in the previous literature, requiring further experiments.

We deduced that the increase in the surface area after the addition of B facilitated carbon diffusion between graphite and austenite, making ferrite formation easier and leading to an increase in the ferrite fraction in the B-added specimens.

### 3.3. Elemental Analysis and SEM/EDS Analysis

The concentration of B in the specimen was confirmed to be 25 ppm by using an inductively coupled plasma optical emission spectrometer (ICP-OES) to analyze the B-added specimen. The concentrations of other alloying elements are also shown in Table 3. To confirm the location of B, energy-dispersive X-ray spectroscopy (EDS) was used to confirm the presence of Cu around the graphite. However, the presence of B was detected at a very low level compared to other elements.

**Figure 5:**
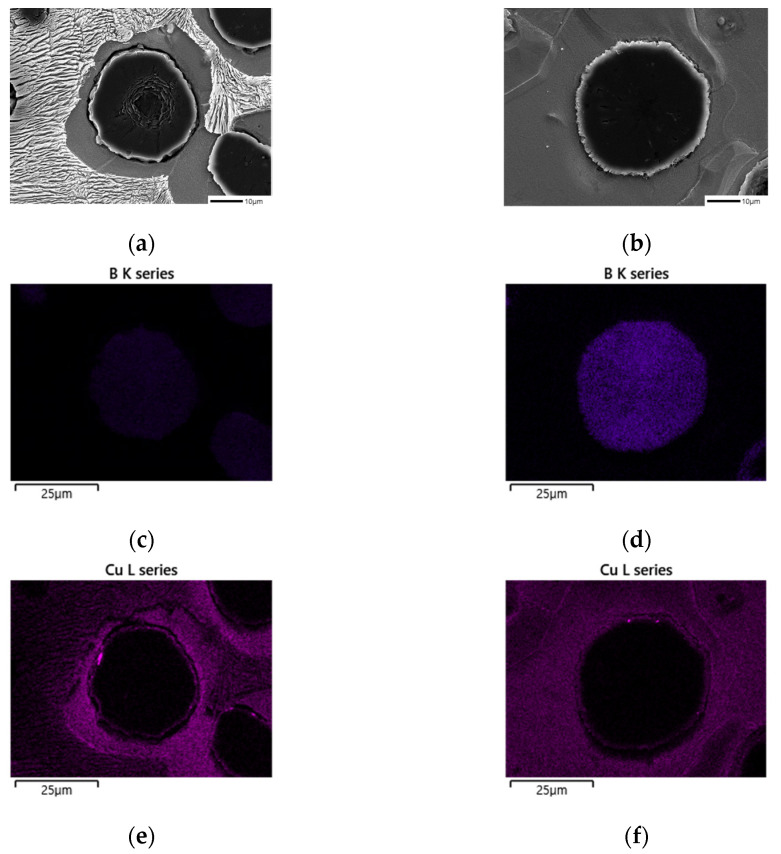
EDS mapping analysis of B and Cu in spheroidal graphite cast iron: (**a**,**c**,**e**) SCI–Mn_0.5_Cu_1_; (**b**,**d**,**f**) SCI–Mn_0.5_Cu_1_B.

Additionally, Figure 5 shows the mapping analysis results for the area around the graphite in the Mn_0.5_Cu_1_B specimen, which qualitatively confirms the presence of B in the graphite region of the B-added specimen. As previously reported, it is expected that B in the graphite forms FeSiBCu, and it is assumed that Cu damages the membrane of the graphite, causing a change in the shape of the graphite [19]. Comparing the Cu distribution in the specimen without B added (a) and the B-added specimen (b) in Figure 5, it can be seen that Cu is more concentrated around the graphite in (a), whereas the distribution of Cu is more evenly spread out in (b).

### 3.4. Thermal Property Analysis

**Figure 6:**
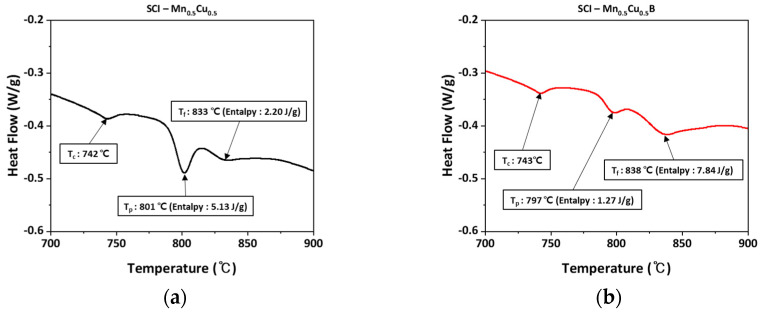
Result from a DSC run for samples with a heating rate of 10 °C/min to 1300 °C: (**a**) SCI−Mn_0.5_Cu_0.5_; (**b**) SCI−Mn_0.5_Cu_0.5_B.

Figure 6 represents a typical composition, and the results of differential scanning calorimetry (DSC) analysis for each composition are presented in Table 4, which includes the following parameters:

T_c_: Curie temperature

T_p_: α + Fe_3_C → γ transformation temperature

T_f_: α → γ transformation temperature

ΔT: T_f_ − T_p_

ΔH_p_: Heat of transformation for α + Fe_3_C → γ transformation

ΔH_f_: Heat of transformation for α → γ transformation

Spheroidal graphite cast iron, also known as ductile iron, undergoes solidification reactions and phase transformations during the heating process. The peak observed during the solidification process is attributed to the transformation of pearlite and ferrite into austenite through carbon diffusion.

When the peak is downward, it indicates a decrease in heat flow, indicating an endothermic reaction. This corresponds to the generation of austenite from pearlite and ferrite due to carbon diffusion, resulting in the observed peak [20,21,22,23].

In SCI–Cu and SCI–CuB with a ferrite fraction of over 90%, the peaks of T_p_ are subtle, while the starting and ending temperatures of T_f_ are similar. However, there is a difference in the value of ΔH_f_.

For SCI–Mn and SCI–MnB specimens, unlike the previous samples, three peaks are observed, which correspond to the Curie temperature, T_f_, and T_p_. The values of ΔH_f_ increase due to the addition of B, while the value of ΔH_p_ decreases.

In Mn_0.5_Cu_0.5_ and Mn_0.5_Cu_1_, which initially had a higher pearlite fraction before the addition of B, the values of ΔH_f_ increase when B is added, while the value of ΔH_p_ decreases, as shown in Table 4.

These results show similar trends to the microstructural findings discussed earlier. While the DSC cannot directly confirm the mechanism of solidification and cooling curves, additional experiments related to the solidification and cooling processes are required since the addition of B did not alter the solidification temperature. However, the increased surface area of graphite observed on the graphite morphology facilitates carbon diffusion, leading to an increase in the enthalpy value of the transforming peaks.

### 3.5. Mechanical Properties Analysis

**Figure 7:**
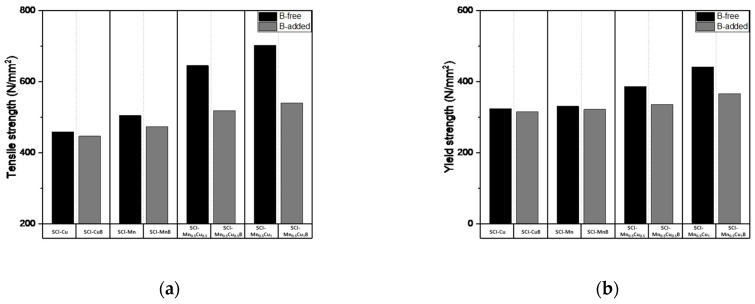
Mechanical properties: (**a**) tensile strength; (**b**) yield strength of specimens.

**Figure 8:**
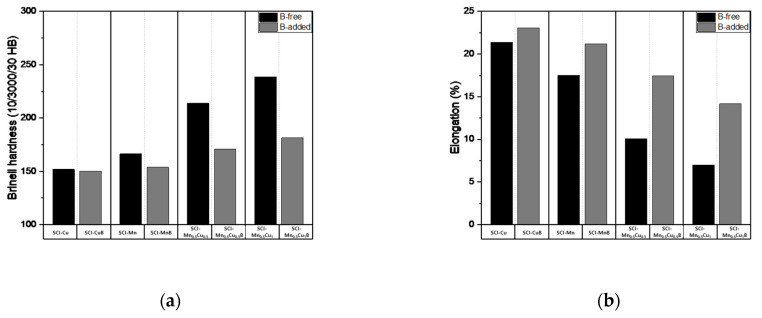
Mechanical properties: (**a**) Brinell hardness; (**b**) elongation of specimens.

**Figure 9:**
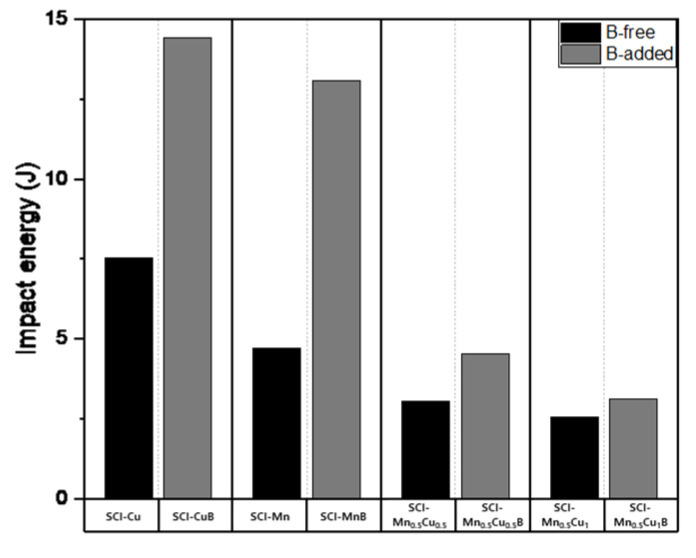
Impact energy of specimens.

The mechanical properties measurement data—including tensile strength, yield strength, hardness, and elongation—of graphite-containing cast iron with and without B are presented in Table 5, and the numerical changes are shown in Figure 7, Figure 8 and Figure 9. Tensile strength shows a decreasing trend with B added, and the effect is more significant when Mn and Cu are co-added, as the increased pearlite fraction due to the addition of these elements has a greater influence. Yield strength and hardness exhibit similar trends to tensile strength. Elongation, which is the opposite of strength, shows an increasing trend with B added, and, similar to other properties, the effect is more pronounced when Mn and Cu are co-added.

These mechanical properties were influenced by the microstructure, as B affected the morphology of graphite, leading to changes in the pearlite and ferrite fractions, which in turn affected the mechanical properties. It can be concluded that the microstructure of the matrix is a critical factor in determining the mechanical properties of graphite-containing cast iron.

The impact energy value showed higher pearlite fraction and elongation values for SCI–MnB than for SCI–Cu, but additional experiments considering the employment characteristics due to Si content will be necessary to explain why the impact energy is lower.

### 3.6. Fracture Surface Analysis

In SCI–Cu specimens, the fracture surfaces were predominantly ductile, as the effect of pearlite formation was minimal, with the pearlite fraction being over 90% even before the addition of B. After B is added, the pearlite fraction increased, resulting in a further increase in ductile fracture.

SCI–Mn specimens generated more pearlite than SCI–Cu specimens, showing a more distinct B addition effect, resulting in a larger ductile area. In addition, the dimple area increased more significantly in SEM measurements.

**Figure 10:**
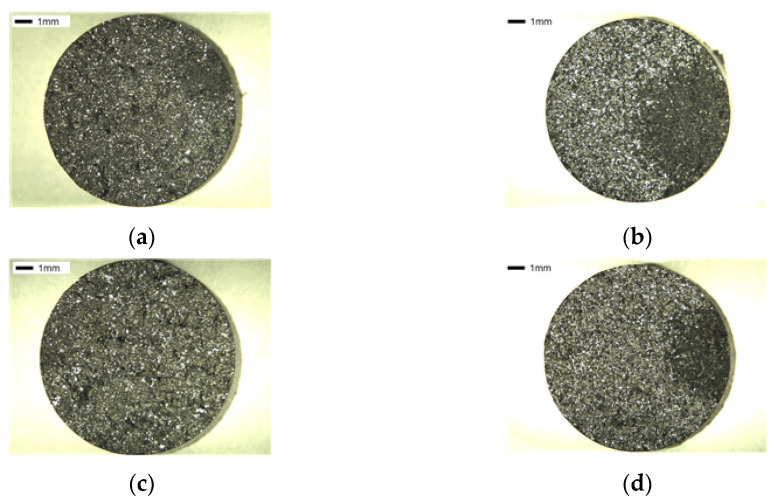
Fraction of specimens: (**a**) SCI–Mn_0.5_Cu_0.5_, (**b**) SCI–Mn_0.5_Cu_0.5_B, (**c**) SCI–Mn_0.5_Cu_1_, and (**d**) SCI–Mn_0.5_Cu_1_B.

The specimens co-added with Mn and Cu had over 60% pearlite fraction, as confirmed in Table 2 and Figure 10, and a clear difference in the increase of ductile area was observed.

## 4. Conclusions

In this study, we investigated the effect of trace amounts of B contained in scrap iron on the properties of spheroidal graphite cast iron (SCI), specifically the changes in microstructure and mechanical properties of the commonly used pearlitic SCI with and without B added.

(1) Copper (Cu) is known as a strong stabilizing element for pearlite, but a small amount of Cu can offset the effects of other pearlitic alloying elements. The addition of Cu with manganese (Mn) was found to be more effective.

(2) The addition of B increased the ferrite fraction in the base structure of SCI, and it showed an increase of up to three times in the maximum ferrite fraction. The effect was more pronounced in pearlitic SCI than in ferritic SCI.

(3) The graphite morphology changed to a surface-enlarging shape after B was added, with increased surface area. Carbon diffusion from austenite during solidification led to increased graphite size and decreased carbon content in austenite, resulting in an increased ferrite area fraction.

(4) Thermal analysis revealed three peaks for the pearlitic SCI in the solidification reaction: the Curie temperature (T_c_), the α + Fe_3_C → γ transformation temperature (T_p_), and the α → γ transformation temperature (T_f_). After B was added, ΔH_p_ decreased while ΔH_f_ increased, indicating an increase in the ferrite area fraction.

(5) After B was added, the tensile strength, yield strength, and hardness decreased, while elongation increased.

(6) The fracture surface showed an increase in ductile fracture area due to the increased ferrite area fraction.

Overall, it can be concluded that the addition of B significantly influences the microstructure and mechanical properties of pearlitic spheroidal cast iron (SCI), where Cu and Mn exhibit distinct effects. Upon interaction with pearlite-forming elements, B addition leads to an increase in ferrite formation heat, resulting in a decrease in strength while simultaneously enhancing ductility. These insights can contribute to the development of high-performance SCI materials with optimized mechanical properties.

In addition, further experiments are needed to investigate the impact of B addition on the impact properties of SCI. The results of impact tests would provide valuable insights into the fracture behavior and toughness of the material. Moreover, additional experiments should be conducted to explore the interaction between B and other pearlite-forming alloying elements in SCI. The effects of different alloying combinations on the microstructure and mechanical properties of the material should be investigated to gain a comprehensive understanding of their synergistic effects. These additional experiments would provide more comprehensive data and a deeper understanding of the influence of B on the properties of pearlitic spheroidal cast iron, facilitating the development of high-performance SCI materials with optimized mechanical properties.

## Figures and Tables

**Table 1:** Target chemical composition of specimens (wt%).

Samples	C	Si	P	S	Mg	Mn	Cu	B
SCI–Cu	3.50	2.60	0.04	0.01	0.045	0.04	0.30	0.0000
SCI–CuB	3.50	2.60	0.04	0.01	0.045	0.04	0.30	0.0025
SCI–Mn	3.50	2.60	0.04	0.01	0.045	0.5	0.00	0.0000
SCI–MnB	3.50	2.60	0.04	0.01	0.045	0.5	0.00	0.0025
SCI–Mn_0.5_Cu_0.5_	3.50	2.60	0.04	0.01	0.045	0.5	0.50	0.0000
SCI–Mn_0.5_Cu_0.5_B	3.50	2.60	0.04	0.01	0.045	0.5	0.50	0.0025
SCI–Mn_0.5_Cu_1_	3.50	2.60	0.04	0.01	0.045	0.5	1.00	0.0000
SCI–Mn_0.5_Cu_1_B	3.50	2.60	0.04	0.01	0.045	0.5	1.00	0.0025

**Table 2:** Resulting microstructure properties of each sample.

Samples	Nodule Count (Count/mm^2^)	Graphite Size (μm)	Graphite Area (%)	Ferrite Ratio (%)
SCI–Cu	196.83	19.60	11.65	92.54
SCI–CuB	186.83	20.41	13.71	94.84
SCI–Mn	259.60	16.83	12.12	73.35
SCI–MnB	203.58	19.65	13.40	82.94
SCI–Mn_0.5_Cu_0.5_	282.55	15.21	11.36	34.15
SCI–Mn_0.5_Cu_0.5_B	264.83	15.87	13.36	65.33
SCI–Mn_0.5_Cu_1_	338.22	14.08	12.26	22.13
SCI–Mn_0.5_Cu_1_B	291.50	16.09	13.58	65.13

**Table 3:** Chemical composition of samples (wt%).

Samples	C	Si	P	S	Mg	Mn	Cu	B
SCI–Cu	3.54	2.68	0.04	0.01	0.04	0.08	0.28	0.0000
SCI–CuB	3.42	2.75	0.04	0.01	0.04	0.08	0.27	0.0029
SCI–Mn	3.37	2.58	0.04	0.01	0.04	0.51	0.02	0.0000
SCI–MnB	3.28	2.60	0.04	0.01	0.03	0.52	0.01	0.0024
SCI–Mn_0.5_Cu_0.5_	3.55	2.53	0.04	0.01	0.04	0.52	0.47	0.0000
SCI–Mn_0.5_Cu_0.5_B	3.50	2.58	0.04	0.01	0.04	0.52	0.48	0.0025
SCI–Mn_0.5_Cu_1_	3.60	2.62	0.04	0.01	0.05	0.56	0.99	0.0000
SCI–Mn_0.5_Cu_1_B	3.45	2.63	0.04	0.01	0.04	0.56	0.99	0.0028

**Table 4:** DSC results of samples. T_c_: Curie temperature, T_p_: α + Fe_3_C → γ temperature, T_f_: α → γ temperature, ΔH_p_: α + Fe_3_C → γ enthalpy, ΔH_f_: α → γ enthalpy.

Samples	T_c_ (°C)	T_p_ (°C)	T_f_ (°C)	ΔT (°C)	ΔH_p_ (J/g)	ΔH_f_ (J/g)
SCI–Cu	748	800	845	45	0.00	6.75
SCI–CuB	747	796	854	59	0.00	11.85
SCI–Mn	746	793	839	46	1.08	7.97
SCI–MnB	746	787	840	53	0.64	8.66
SCI–Mn_0.5_Cu_0.5_	742	801	833	32	5.13	2.20
SCI–Mn_0.5_Cu_0.5_B	743	797	838	41	1.27	7.84
SCI–Mn_0.5_Cu_1_	739	800	835	35	8.67	0.91
SCI–Mn_0.5_Cu_1_B	739	797	835	38	1.85	4.06

**Table 5:** Mechanical properties of samples.

Samples	TS (N/mm^2^)	E (%)	YS (N/mm^2^)	HB (10/3000/30)	Impact Energy (J)
SCI–Cu	459.28	21.38	324.36	152.00	7.53
SCI–CuB	446.93	23.07	315.36	150.00	14.43
SCI–Mn	504.89	17.51	331.17	166.33	4.70
SCI–MnB	473.77	21.19	322.46	153.75	13.09
SCI–Mn_0.5_Cu_0.5_	645.49	10.08	386.46	214.00	3.05
SCI–Mn_0.5_Cu_0.5_B	518.62	17.45	335.74	171.00	4.54
SCI–Mn_0.5_Cu_1_	703.15	7.01	442.05	238.67	2.55
SCI–Mn_0.5_Cu_1_B	539.95	14.16	366.02	181.33	3.13

## Data Availability

The data presented in this study are available on request from the corresponding author and the first author.
